# Early-Life Social Isolation Impairs the Gonadotropin-Inhibitory Hormone Neuronal Activity and Serotonergic System in Male Rats

**DOI:** 10.3389/fendo.2015.00172

**Published:** 2015-11-10

**Authors:** Tomoko Soga, Chuin Hau Teo, Kai Lin Cham, Marshita Mohd Idris, Ishwar S. Parhar

**Affiliations:** ^1^Brain Research Institute, School of Medicine and Health Sciences, Monash University, Selangor, Malaysia

**Keywords:** GnRH, dorsomedial hypothalamus nuclei, serotonin, social stress, dorsal raphe nuclei

## Abstract

Social isolation in early life deregulates the serotonergic system of the brain, compromising reproductive function. Gonadotropin-inhibitory hormone (GnIH) neurons in the dorsomedial hypothalamic nucleus are critical to the inhibitory regulation of gonadotropin-releasing hormone neuronal activity in the brain and release of luteinizing hormone by the pituitary gland. Although GnIH responds to stress, the role of GnIH in social isolation-induced deregulation of the serotonin system and reproductive function remains unclear. We investigated the effect of social isolation in early life on the serotonergic–GnIH neuronal system using enhanced green fluorescent protein (EGFP)-tagged GnIH transgenic rats. Socially isolated rats were observed for anxious and depressive behaviors. Using immunohistochemistry, we examined c-Fos protein expression in EGFP–GnIH neurons in 9-week-old adult male rats after 6 weeks post-weaning isolation or group housing. We also inspected serotonergic fiber juxtapositions in EGFP–GnIH neurons in control and socially isolated male rats. Socially isolated rats exhibited anxious and depressive behaviors. The total number of EGFP–GnIH neurons was the same in control and socially isolated rats, but c-Fos expression in GnIH neurons was significantly reduced in socially isolated rats. Serotonin fiber juxtapositions on EGFP–GnIH neurons were also lower in socially isolated rats. In addition, levels of *tryptophan hydroxylase* mRNA expression in the dorsal raphe nucleus were significantly attenuated in these rats. These results suggest that social isolation in early-life results in lower serotonin levels, which reduce GnIH neuronal activity and may lead to reproductive failure.

## Introduction

Gonadotropin-releasing hormone (GnRH) and the newly identified neuropeptide gonadotropin-inhibitory hormone (GnIH) are regulators of reproductive activity in vertebrates (1). GnIH neuropeptides contain an Arg–Phe–NH_2_ motif [LPXRFamide (X = L or Q) sequence] at their C termini in most vertebrate species. Rat LPXRFamide peptides are known as RFamide-related peptides (RFRPs; RFRP-1 and -3) (2). GnIH-expressing cells are mainly located in the dorsomedial hypothalamic nucleus (DMN) in rats (3, 4) and send fiber projections to GnRH neurons in the pre-optic area (POA), median eminence, and other areas of the brain in rodents (5–8). GnIH acts on GnRH neurons through its seven-transmembrane domain G protein-coupled receptor (GPR) 147 (7, 9).

Recent studies have demonstrated that GnIH neuronal activity is linked to the hypothalamic–pituitary–adrenal (HPA) axis, since glucocorticoids can stimulate *gnih* mRNA expression (10–12). Our recent study (8) showed that exposure to the glucocorticoid receptor agonist dexamethasone during early life increases GnIH expression, GnIH receptor expression, and the number of fiber projections to the POA in adult female mice. These findings suggest that the GnIH system is sensitive to glucocorticoids, which can influence GnRH neuronal activity and reproduction.

Stress and glucocorticoids modulate the serotonin [5-hydroxytryptamine (5-HT)] system in the brain. Several lines of evidence suggest that 5-HT can modulate GnIH neuronal activity. Cellular localization of 5-HT_1A_ receptors is evident in the DMN, where GnIH neurons reside in the rat brain (13). In fact, 5-HT receptors (5-HT_1A, 1B, 1D, 1F, 2A, 2B, 3A, 5A, 5B, 6, and 7_) are co-expressed in GnIH neurons in the DMN (14). Acute glucocorticoid treatment can cause 5-HT to accumulate in the DMN (15, 16) and treatment with the antidepressant citalopram can stimulate the GnIH system in male mice (14). The 5-HT system of the brain modulates sexual behavior, sexual arousal, and motivation in rodents (17); thus, 5-HT–GnIH signaling may participate in the negative regulation of reproductive activity, including sexual behavior.

Social isolation, a passive stress, activates the HPA axis (18, 19), causes imbalances in 5-HT turnover in rats (20) and decreases the number of 5-HT neurons, expression of 5-HT receptors, number of 5-HT fiber projections in the hippocampus (21–23) and binding activity of the 5-HT_1A_ receptor (24). Indeed, social isolation in early life impairs 5-HT-associated functions of the brain, which include the control of anxiety, depression, aggression (22, 25–27), and sexual behavior in adult male rats (28–30). Furthermore, social isolation results in a significantly higher level of plasma testosterone (31, 32) and increased testis weight (32) in male rats. Social isolation in early life may affect the production of sex steroids and related behaviors, such as sexual behavior and aggression. The neuronal mechanism underlying early-life social isolation-induced reproductive failure through the deregulation of 5-HT and the GnIH system remains unknown.

We examined the activity of GnIH neurons in our newly created enhanced green fluorescent protein (EGFP)–GnIH transgenic rats. We measured 5-HT fiber juxtapositions to and c-Fos protein expression in GnIH neurons using immunohistochemistry and mRNA levels of the 5-HT associated genes, *serotonin transporter* (*sert*) and *tryptophan hydroxylase 2* (*tph2*), using real-time polymerase chain reaction (PCR) in male rats socially isolated in early life. The examination of this model of social isolation in early life may improve our understanding of mental disorders, as well as sexual dysfunction caused by passive stress, in young adults.

## Materials and Methods

### Animals

Male transgenic Wistar rats expressing enhanced green fluorescent protein (EGFP) under rat GnIH promoter (GnIH–EGFP transgenic rats) (4), after weaning (3 weeks of age), were randomly assigned to group housing (2–4 male littermates per cage) (*n* = 63) or individual housing (isolated) condition (*n* = 65) up to 9 weeks of age. The animals were maintained under a controlled 12 h light/dark cycle (lights on from 12:00 a.m. to 12:00 p.m.) with temperature maintained at 22°C in the SPF animal facility for 6 weeks prior to sampling. Autoclaved water and food were available *ad libitum* to the rats. Body weight of each rat was measured once a week. All aspects of animal welfare and experiments were in accordance with the guidelines and authorization of Monash University Animals Ethics Committee, AEC (MARP/2012/140, MARP/2013/041).

### Anxiety and Depression-Like Behavior Tests

The open field test (OFT) was conducted in an open field area made up of a black box (width: 1.2 m, length: 1.2 m, and height: 0.30 m) for use with white rats of 9 weeks old. A handheld camcorder (Sony Corp., Japan) was used to record the movement of the rat in the arena. The OFT experiments were performed in light (white lighting, 10:00 a.m.–12:00 p.m.) and dark conditions (red lighting, 3:00–6:00 p.m.). The video file from the camcorder was analyzed using an automated motion detector software (Lolitrack v2.0, Loligo Systems, Denmark) to track the movements of the rat. Control rats (group housed; *n* = 11/light phase and *n* = 9/night phase) and isolated rats (*n* = 12/light phase and *n* = 13/night phase) were subjected to OFT for 30 min in order to observe anxiety-like behavior in the dark phase and light phase. The total distance traveled (cm), total time of activity (s), total number of center intrusions, and total time spent in center (s) were measured.

Forced swimming test (FST) was carried out using an automated behavior analytical system (MicroAct system, Neuroscience, Inc., Tokyo, Japan). FST was carried out twice; a pre-test as a habituation session and the actual test was performed 24 h later. The FST apparatus consisted of a glass cylinder (height: 45 cm and diameter: 20 cm) which was surrounded by round coil. Prior to the FST, the glass cylinder was filled with water (25 ± 1°C) to a depth of 30 cm, and a magnet (diameter: 1 mm and length: 3 mm) was taped to each front paw of the rats. Individual rat was gently lowered into the water-filled glass cylinder for a 7 min swim test. Electrical currents were generated in the coils corresponding to the movements of the magnet taped to the front paws. The currents were amplified, transformed into voltage, and recorded by the system. The duration of immobility was detected automatically using the *MicroAct*™ Scratch software (Neuroscience, Inc., Tokyo, Japan). Twenty-six male rats were randomly allocated to group-housing conditions (*n* = 17) or isolated conditions (*n* = 9) and used in the FST.

The sucrose preference test (SPT) was conducted over a period of 5 days. Prior to the test, the rats were habituated to the presence of two water bottles in their cages for a minimum of 5 days beforehand. The rats undergoing the experiment were provided with two water bottles: one containing sucrose water (sucrose powder diluted in distilled water, 200 mL) and the other containing only distilled water (200 mL). The concentration of the sucrose water was increased every day over a period of 5 days (0, 0.25, 0.5, 1.0, and 2.0%). Water consumption was measured at 12:00 p.m. everyday by weighing the water bottles to determine consumption by weight. The position of the water bottles was swapped daily at the time of measurement to reduce preference bias in the results. The data for percentage of sucrose consumed against total water consumed was calculated from the results and used as an indicator for sucrose preference. A repeated measures test was used for statistical analysis in order to confirm any significant difference between group-housed and isolated rats in their pattern of sucrose preference over the 5-day test period. Thirty-one male rats were randomly allocated to group-housing conditions (*n* = 14) or isolated conditions (*n* = 17) at 9 weeks of age. We divided the control and the post-weaning social isolation rats each into two groups for three behavioral tests. Group I was used for OFT and FST and group II was tested for SPT.

### Polymerase Chain Reaction

Control (*n* = 6) and socially isolated rats (*n* = 6) at 9 weeks of age were deeply anaesthetized with an intraperitoneal injection of ketamine xylazine (4.5 mg/kg/BW) followed by rapid removal of the brain and immediately dissected by 1 mm rat brain slicer (Neuroscience, Inc., Japan). The POA (bregma +1.2 to −0.12) and dorsal raphe (bregma −6.96 to −8.16) areas were collected with a sterile blade. Total RNA from these tissues was extracted using TRIzol (Invitrogen, Carlsbad, CA, USA) and transcribed using High Capacity Transcription Kit (Applied Biosystems, Foster City, CA, USA) according to the manufacturer’s protocols. Quantitative real-time PCR (ABI 7300, Applied Biosystems Foster City, CA, USA) was performed using primers for *gnrh*, *gnih*, *sert*, *tph*, and *IMPDH2* (0.2M, Table S1 in Supplementary Material) in a final volume of 10 μl of 2X Power SYBR Green PCR mix (Applied Biosystems). The house keeping gene, *IMPDH2* is listed as a reference gene in real-time PCR to show geometric average expression level (33). The resulting PCR products were validated using an ABI PRISM 310 Genetic Analyzer and Sequence Analysis Software (Applied Biosystems) and ran on a 2.5% agarose gel with ethidium bromide used for visualization.

### Immunohistochemistry

Immunocytochemistry for cFos, 5-HT, and 5-HT_2A_ was performed on the DMN sections obtained through coronal sectioning (30 μm). The perfusion fixed (4% PFA) brain tissue sections were washed with 0.1M PBS, in an incubation chamber for 10 min at room temperature and gently shaken at 60 rpm. The sections were then incubated in a blocking solution (40 μL normal goat serum (NGS), 10 μl 0.5% Triton-X, and 1950 μl PBS in 2 mL/well) for 1 h in the same conditions as above. After washing, the sections were incubated with polyclonal rabbit anti-c-Fos antiserum diluted 1:600 (sc52, Santa Cruz Biotechnology, Inc., USA), goat anti-5HT antiserum diluted 1:1000 (20079, Immunostar Inc., WI, USA), rabbit anti-5HT_2A_ antiserum diluted 1:200 (24288, Immunostar Inc., WI, USA) in 2 mL 0.1M PBS containing 2% NGS, 0.5% Triton-X/well for 24 h at 4°C for c-Fos, 5-HT and 5-HT_2A_ respectively at 4°C. Next, the sections were washed in 0.1M PBS incubated in biotinylated anti-rabbit immunoglobulin G (IgG) or biotinylated anti-goat IgG (Vectastain ABC Elite kit, Vector Laboratories, Burlingame, CA, USA) for 45 min. Subsequently, the sections were incubated with avidin-biotinylated horseradish peroxidase complex for 45 min (Vectastain ABC Elite kit, Vector laboratories, Burlingame, CA, USA). Sections were visualized with Alexa Fluor 594 streptavidin conjugates (S32356, Invitrogen Corporation, USA) and pasted on microscope slides (Superfrost PLUS, Fisher Scientific, Pittsburgh, PA, USA). Mounting medium was applied (VectaShield, Vector Laboratories) followed by coverslips. The number of immunoreactive EGFP–GnIH cells within the DMN were determined using the laser scanning confocal microscope (C1si, Nikon, Tokyo, Japan), equipped with NIS-Element 4.0 Advance software. The specificity of both c-Fos and 5-HT antibody was tested using the rat brain from previous study (4). We divided the control and the post-weaning social isolation rats each into two groups. Group I was used for c-Fos, 5-HT (control: *n* = 20, isolated condition: *n* = 17) and group II was tested for 5-HT_2A_ (*n* = 3). Double-labeled images of c-Fos and GnIH staining, viewed under the red channel were converted to magenta. The brightness and the contrast were adjusted using Adobe Photoshop CS2 (Adobe, San Jose, CA, USA).

### Confocal Analysis of c-Fos Expression and 5-HT Fiber Juxtapositions to GnIH Neurons

The procedure for confocal analysis of c-Fos expression in GnIH neurons has been described previously (4). Briefly, immunoreactive c-Fos positive GnIH neurons were visualized using digitized images captured with a Nikon-30 confocal microscope (C1si, Nikon Instruments Inc., Tokyo, Japan). The total number of GnIH neurons and immunoreactive c-Fos positive GnIH neurons were determined using 0.225 μm Z-steps in 10–15 sections which included all EGFP–GnIH neurons in the DMN. To confirm the co-localization of c-Fos in GnIH neurons, the Z-steps were carefully inspected with 3D image rotation using NIS Elements AR Version 4.0 (Nikon Instruments Inc.). We then calculated the percentage of c-Fos positive GnIH neurons. Only cells with visible nuclei were counted. The procedure for confocal analysis of fiber projections to GnIH neurons has been described previously (4). Briefly, 5-HT fiber juxtapositions were captured with a confocal microscope at 0.225 μm Z-steps using 60× water immersion objective lens, 4× digital zoom function to cover the entire depth of the neuron (ECLIPSE 90i, Nikon instruments Inc., Japan). Scans of 488 and 543 nm excitation wavelength were also performed sequentially across optical sectioning to avoid bleed-through between the channels. The number of GnIH neurons with intimate 5-HT fiber juxtapositions was determined in 10–15 sections to include all EGFP–GnIH neurons in the DMN. To confirm close juxtapositions between 5-HT fibers and GnIH neurons, the Z-steps were carefully inspected with 3D rotation image using NIS Elements AR Version 4.0 (Nikon Instruments Inc.). GnIH neurons with 5-HT fiber juxtapositions on the cell soma or dendrites were counted. A contact was scored only if 5-HT fiber varicosity was in direct contact with the GnIH neuron. The percentage of GnIH neurons with visible nuclei in the DMN and with at least one close juxtapositions with 5-HT fiber was calculated.

### Statistics

Data are presented as means ± SEM in all bar graphs. Behavioral data were analyzed by two-way apposition using SPSS 20 (IBM, Chicago, IL, USA). SPT was analyzed by a univariate repeated measures using SPSS 20. Immunohistochemistry and gene expression results were analyzed using the Student’s *t*-test. Significance was set as *p* < 0.05.

## Results

### Social Isolation, Anxiety, Depression, and the Serotonin System

**Figure 1:**
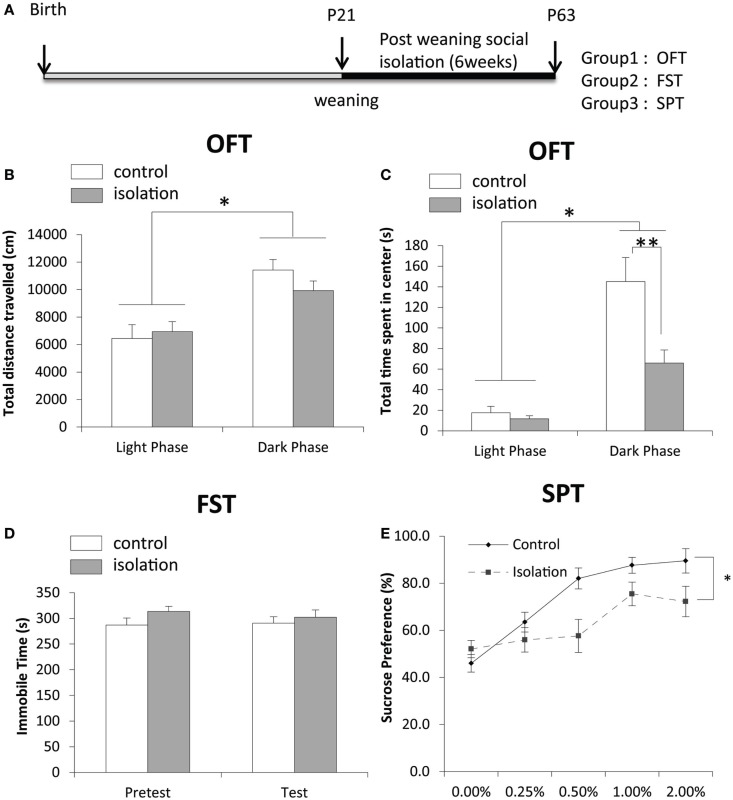
**The effect of post-weaning social isolation on behavior**. **(A)** Experimental timeline of social isolation and sampling for four experiments. **(B)** Total distance traveled in an open field test (OFT) in the light (CT8–10) and dark (CT15–18) phases in control and socially isolated rats (light-phase control: *n* = 11, light phase isolated: *n* = 12, dark-phase control: *n* = 9, dark-phase isolated: *n* = 13). **(C)** Total time spent in the center during the OFT (s) in the light and dark phases in control and socially isolated rats. **(D)** Time spent immobile during a forced-swim test for 7 min (control: *n* = 9 and isolated: *n* = 17). **(E)** Comparison of sucrose preference was expressed as a percentage of consumption in control (*n* = 14) and isolated (*n* = 17) rats. Data are presented as mean ± SEM for each set. *p < 0.05 and **p < 0.01.

After 6 weeks of social isolation, we conducted three behavioral tests and took samples of brain tissues for biological study (Figure 1A). Although the total distance traveled (cm), total time of activity (s), total number of intrusions into the center, and total time spent in the center (s) were not significantly different between control and isolated rats, both control and isolated rats traveled more and were active for longer durations in the dark phase compared with the light phase (Figure 1B). No significant difference in activity in the light phase was evident between control and isolated animals (Figure 1B). However, the total time spent in the center by socially isolated rats was significantly shorter in the dark phase than in controls (control: 145.11 ± 23.28 s, isolated: 65.85 ± 12.73 s, *p* < 0.05; Figure 1C). Control (*n* = 17) and isolated (*n* = 9) rats were subjected to a forced-swim test for 7 min to measure time spent immobile. A pre-test was conducted 24 h earlier for habituation. No significant difference was observed in time spent immobile between control and isolated rats during the pre-test or test sessions (Figure 1D). The difference in sucrose consumption between groups was measured over 5 days. Using a univariate repeated measures test for analysis, a significant difference was observed in sucrose preference between control and isolated rats [control: *F*(1,31) = 6.168, *p* < 0.05; Figure 1E].

### Social Isolation and Reproduction

**Figure 2:**
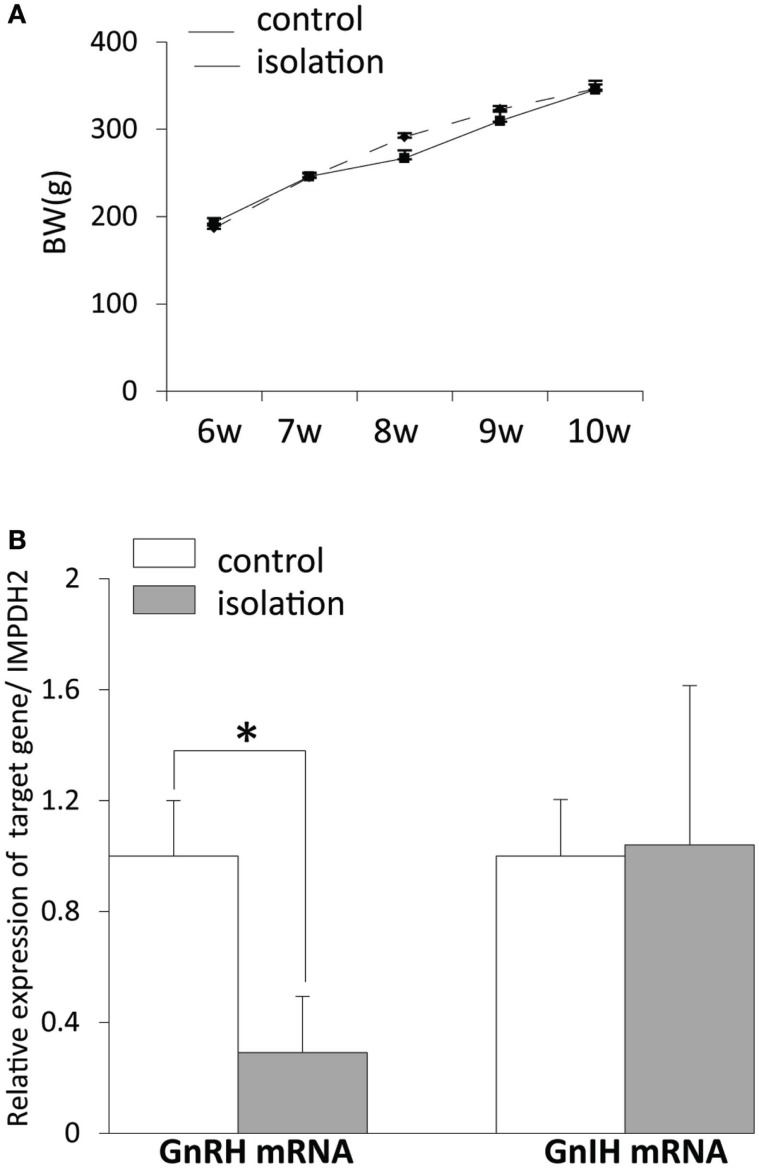
**The effect of post-weaning social isolation on body weight and reproductive neuropeptide gene expression in the brain**. **(A)** Body weight changes during post-weaning social isolation (*n* = 6/housing). **(B)** Post-weaning social isolation decreased the levels of *gonadotropin-releasing hormone* mRNA in the pre-optic area (*n* = 6/housing) but did not change the levels of *gonadotropin-inhibitory hormone* mRNA in the hypothalamus (*n* = 6/housing). The relative mRNA expression levels were normalized to that of *inosine 5*′*-monophosphate dehydrogenase 2* mRNA. All data are presented as mean ± SEM. Significant differences were determined using the Student’s *t*-test for unpaired values; significance was set at *p < 0.05.

There was no difference in body weight post-weaning between controls and socially isolated male rats (Figure 2A). The level of *gnrh* mRNA in the POA was significantly lower in socially isolated rats compared with controls (control: 1 ± 0.2 and isolated: 0.29 ± 0.2, *p* < 0.05; Figure 2B). However, there was no difference in the expression of *gnih* mRNA in the hypothalamus between socially isolated and control rats (Figure 2B).

### Social Isolation and Gonadotropin-Inhibitory Hormone Neuronal Activity

**Figure 3:**
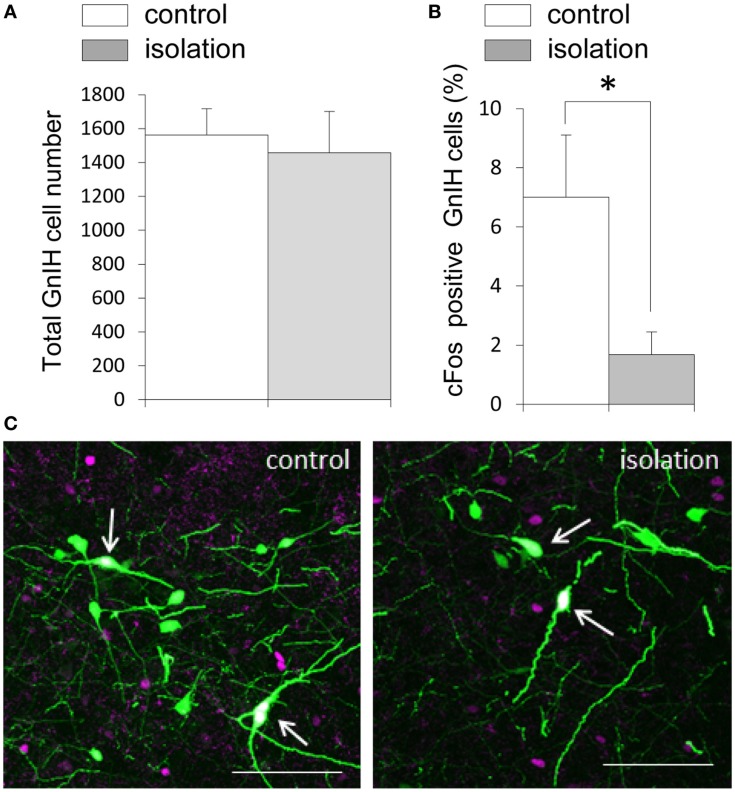
**The effect of post-weaning social isolation on enhanced green fluorescent protein–gonadotropin-inhibitory hormone neurons in the dorsomedial hypothalamic nucleus**. **(A)** Total number of gonadotropin-inhibitory hormone (GnIH) cells in the dorsomedial hypothalamic nucleus (DMN) of control and isolated male rats (control = 14 and isolated = 11). **(B)** Percentage of c-Fos-positive GnIH neurons in the DMN of control and isolated male rats. Data represent the mean ± SEM for each group. *p < 0.05. **(C)** Confocal images of enhanced green fluorescent protein (EGFP)–GnIH cells expressing c-Fos protein (white, indicated by arrows), EGFP–GnIH neurons (green), and red c-Fos protein (magenta) in control (left panel) and isolated (right panel) male rats. Scale bar: 100 μm.

EGFP–GnIH cell bodies were visible in the DMN, which comprised the central, ventral, and dorsal portions of the DMN, and in the dorsal tuberomammillary nucleus (DTM). There was no significant difference in the total number of EGFP–GnIH cells in the entire DMN and DTM between isolated males and group-housed control males [control: 1561.43 ± 156.72 (*n* = 14) and isolated: 1457.55 ± 244.48 (*n* = 11); Figure 3A]. To study the effect of social isolation on GnIH neuronal activity, c-Fos immunoreactivity was analyzed in GnIH neurons using a laser scanning confocal microscope. The percentage of GnIH cells exhibiting c-Fos immunoreactivity in the entire DMN was significantly decreased in isolated males compared with group-housed control males (control: 7.01 ± 2.2% and isolated: 1.68 ± 0.77%, *p* < 0.05; Figures 3B,C).

### Social Isolation and Serotonergic Regulation of Gonadotropin-Inhibitory Hormone Cells

**Figure 4:**
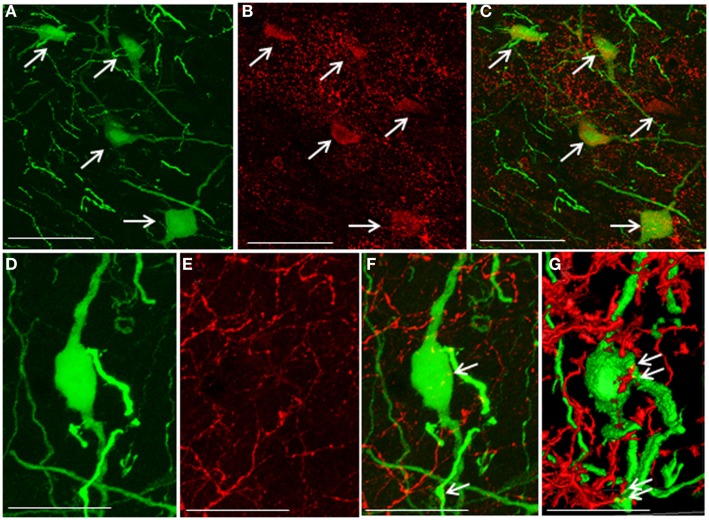
**Co-localization of 5-hydroxytryptamine fibers and the 5-hydroxytryptamine_2A_ receptor in enhanced green fluorescent protein–gonadotropin-inhibitory hormone cells in the dorsomedial hypothalamic nucleus**. **(A)** Enhanced green fluorescent protein (EGFP)–gonadotropin-inhibitory hormone (GnIH) cells and fibers (green), **(B)** 5-hydroxytryptamine _2A_ (5-HT_2A_)-immunostained neurons (red), and **(C)** EGFP–GnIH cells expressing 5-HT_2A_ (yellow/orange) and 5-HT_2A_-immunostained neurons (red) in the dorsomedial hypothalamic nucleus (DMN). **(D)** EGFP–GnIH neurons (green), **(E)** 5-hydroxytryptamine (5-HT)-immunostained fibers (red), and **(F)** 5-HT in close juxtapositions in EGFP–GnIH neurons or fibers (yellow dot, indicated by arrows). **(G)** 3D images of 5-HT in close juxtapositions in EGFP–GnIH neurons or fibers (yellow dot, indicated by arrows) in the DMN. Scale bars: **(A–C)**, 50 μm; **(D–G)**, 20 μm.

**Figure 5:**
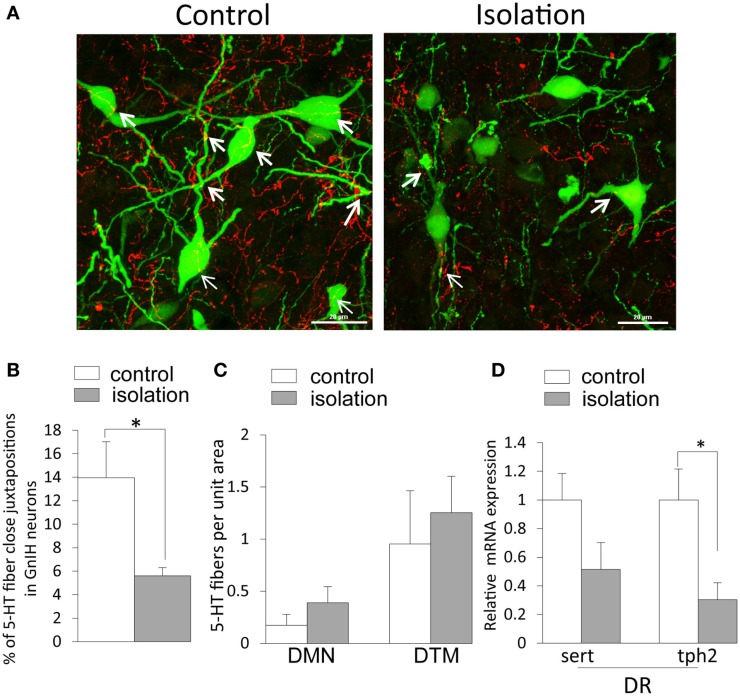
**The effect of post-weaning social isolation on 5-hydroxytryptamine projections in enhanced green fluorescent protein–gonadotropin-inhibitory hormone neurons in the dorsomedial hypothalamic nucleus and 5-hydroxytryptamine-related gene expression in the dorsal raphe nucleus**. **(A)**. Confocal images of gonadotropin-inhibitory hormone (GnIH) cells expressing 5-hydroxytryptamine (5-HT) fiber juxtapositions (enhanced green fluorescent protein–GnIH neurons, green; 5-HT, red; fiber juxtapositions; yellow indicated by arrows) in control (left panel) and isolated (right panel) male rats. Scale bar: 20 μm. **(B)** Percentage of 5-HT fibers in close juxtapositions in GnIH cells in the dorsomedial hypothalamic nucleus (DMN) of control and isolated male rats (control = 6 and isolated = 6). **(C)** 5-HT immunostaining density per unit area in the DMN and dorsal tuberomammillary nucleus of control and isolated male rats. **(D)** Relative *serotonin transporter* and *tryptophan hydroxylase 2* mRNA expression was normalized to that of the housekeeping gene *inosine 5*′*-monophosphate dehydrogenase 2* mRNA in the dorsal raphe nucleus of control and socially isolated rats (*n* = 6/housing). Data are presented as mean ± SEM for each group. *p < 0.05.

5-HT_2A_-positive cells were evident in the DMN. Some EGFP–GnIH neurons co-expressed 5-HT_2A_ (Figures 4A–C). In addition, close juxtapositions between 5-HT-immunoreactive fibers and GnIH cell bodies were observed in the DMN (Figures 4D–G). To study the effect of social isolation on the serotonergic regulation of GnIH cells, close juxtapositions between 5-HT-immunoreactive fibers and GnIH cell bodies was determined and analyzed using a laser scanning confocal microscope. The percentage of 5-HT-immunoreactive fibers in close juxtapositions to GnIH cells in the entire DMN and DTM was significantly decreased in isolated males compared with group-housed control males (control: 13.96 ± 3.05% and isolated: 5.6 ± 0.7%, *p* < 0.05; Figures 5A,B). However, 5-HT fiber density per unit area in the DMN was the same in control and isolated rats (Figure 5C). Expression of the 5-HT-related genes *sert* and *tph2* in the dorsal raphe nucleus (DR) was measured using quantitative real-time PCR. There were no differences in the levels of *sert* mRNA expression between control and socially isolated rats. However, *tph2* mRNA expression was significantly lower in socially isolated rats (*n* = 6) compared with controls (*n* = 6; control: 1 ± 0.22 and isolated: 0.3 ± 0.11, *p* < 0.05; Figure 5D).

## Discussion

In this study, we showed that post-weaning social isolation impairs GnIH neuronal activity and the serotonergic system in the DMN, which may contribute to the deregulation of GnRH neuronal activity in the POA and sexual dysfunction.

### Social Isolation, Behavior, and the Serotonin System

It is established that post-weaning isolation for 6 weeks from post-natal day (P) 21 (the time of weaning) has serious consequences for brain development, causing alterations in neurotransmission and behavioral abnormalities (aggression, anxiety, and depression) in rodents (20, 34, 35). This suggests that social stimuli received after weaning are critical to the development of social behaviors and related neuronal circuits. We observed a daily variation in anxiety-like behavior in both group-housed and socially isolated rats. Importantly, during the dark phase, anxiety-like behaviors were observed in socially isolated rats. However, total locomotor activity was unaffected by group or socially isolated housing. These data suggest that the anxiogenic effect of post-weaning social isolation could depend on light conditions and their effect on circadian rhythm. Indeed, disrupted sleep patterns are evident in socially isolated rats (36).

The total time spent immobile (a parameter of depressive-like behavior) is unaffected by post-weaning social isolation in rats (37). Several studies have shown that social isolation increases despair-like immobility (38–40) and immobile time in male rats (41). These conflicting results may be explained by differences between rat strains and the duration of social isolation. Furthermore, in this study, post-weaning isolation resulted in a decreased sucrose intake and reduced preference for sucrose. This anhedonia-like phenotype induced by social isolation can be reversed by treatment with the antidepressant imipramine in male rats (30), suggesting that it is mediated by the 5-HT pathway. For the first time, we show that post-weaning social isolation specifically decreases 5-HT fiber projections to the DMN; this may, in turn, cause the downregulation of *sert* gene expression in the DR, where 5-HT neurons are primarily located. This is supported by the decrease in central 5-HT and 5-HT receptors evident in socially isolated animals during episodes of increased anxiety (27, 42). Although evidence of serotonergic and 5-HT receptor activity in the DMN remains inconclusive, serotonergic projections to DMN neurons have been reported (43). Indeed, in this study, we found that 5-HT_2A_ is co-localized in GnIH neurons in rats. Although the magnitude of changes in 5-HT_2C_ in GnIH neurons in socially isolated rats is unknown, antagonists of 5-HT_2C_ receptors reportedly increase sucrose preference (44). Several 5-HT receptor types, including 5-HT_2C_, are expressed in GnIH neurons in the DMN of female mice (8). Therefore, the alteration of serotonergic signaling in the DMN may underlie the reduced preference for sucrose in socially isolated rats. Therefore, GnIH neurons and other neurons in the DMN may be targets of the circuitry for anxiety and anhedonia that mediates serotonergic activity following post-weaning social isolation.

### Social Isolation and Reproduction

Reproductive senescence can be caused by factors related to the social environment. Stress in early life delays pubertal onset, lowers GnRH expression, lowers testosterone synthesis, and impairs sexual behavior, all of which eventually lead to sexual dysfunction in mammals (31, 32, 45–47). Post-weaning social isolation impairs male sexual behavior, as indicated by an increased latency of ejaculation during adulthood (30, 48). GnRH expression and release from the POA is a key regulator of gonadotropin release and reproductive behavior. Our results show that post-weaning social isolation decreases the expression of GnRH mRNA in male rats, which could lead to sexual dysfunction.

### Social Isolation and Gonadotropin-Inhibitory Hormone Neuronal Activity

This study is the first to demonstrate GnIH neuronal activity following post-weaning social isolation. Although it is established that GnIH inhibits GnRH neuronal activity (7, 9) and GnRH induced-LH release by the pituitary gland (5), we found that post-weaning social isolation decreases both GnRH mRNA expression and GnIH neuronal activity. Thus, both the GnRH and GnIH systems are down regulated following post-weaning social isolation. In rats, neural activity and the release of GnRH are increased just before and after puberty in the POA (49), which coincides with pubertal processes, such as changes in γ-aminobutyric acid (GABA) and glutamate levels (50). Morphological changes, such as structural remodeling of the dendrites of GnRH neurons, are the key change during puberty (51). Although the timing of the formation of inhibitory GnIH neuronal inputs to GnRH neurons during the post-natal period remains unknown, a lack of social stimuli pre- and post-puberty may have an impact on the formation of GnIH inputs to GnRH neurons. Post-weaning social isolation may disturb normal GnIH–GnRH neuronal signaling during pubertal development, which may result in reduced expression of GnRH and GnIH in the brain.

Accumulated evidence from recent studies (8, 10, 12) shows that stress increases GnIH expression, with an associated suppression of the hypothalamic–pituitary–gonadal axis suggesting that the inhibitory effect of stress on reproductive function may be mediated by the GnIH system. Post-weaning social isolation causes hypofunction of the HPA axis in adult rats, suggesting that the HPA axis becomes desensitized to stressful stimuli (52). GnIH neurons are sensitive to stress (10–12); thus, in socially isolated rats, they may also become desensitized, as implied by their low neuronal activity. Long-term social isolation lowers GnRH mRNA expression in the POA during adulthood; therefore, inhibitory GnIH signaling may be reduced as a result of short-loop negative feedback from GnRH neurons. Post-weaning social isolation may disrupt the normal development and balance of the GnIH–GnRH neuronal pathway for reproductive activity.

We did not observe any erroneous positioning of EGFP–GnIH neurons in the DMN of socially isolated rats. Likewise, social isolation had no effect on the total number of EGFP–GnIH cells. During development, GnIH expression starts at embryonic days (E) 13–14. GnIH neurons migrate to the dorsal and ventral regions of the third ventricle at E16–17 and establish their positions in the medial hypothalamus by E18 (53, 54). GnIH neurons send ascending and descending projections to other regions of the brain by P1 and the GnIH neuronal system is almost completely formed by P28 (3, 53). Our study shows that the development and positioning of GnIH neurons in the DMN during the prenatal period is not altered by post-weaning (after P21) social isolation.

### Social Isolation and the Serotonergic Regulation of Gonadotropin-Inhibitory Hormone Neurons

5-HT-immunoreactive fibers form close juxtapositions to GnIH neurons in the DMN of the rat brain. The DMN receives 5-HT fibers and terminals through the medial forebrain bundle from 5-HT nerve cell bodies of the DR (55). Our findings related to 5-HT_2A_, and those of our previous study (14), show that 5-HT receptors are co-expressed in GnIH neurons in female mice. Additionally, administration of citalopram increases the number of GnIH neurons in the DMN and the density of GnIH fibers in the POA (14), supporting the concept that GnIH is under direct serotonergic control. The significant decrease in c-Fos expression evident in GnIH neurons, combined with the decreased 5-HT innervation of GnIH neurons in socially isolated animals, suggest a reduction in GnIH neuronal activity. Early environmental manipulations impair long-term synaptic potentiation (56). The decrease in 5-HT fiber juxtapositions to GnIH neurons reflects neuroanatomical adaptation of hypothalamic neuronal circuits in response to a deprived social environment in early life. 5-HT synaptogenesis is important for brain 5-HT concentration during the critical period of brain development (57). Moreover, 5-HT receptor complements are established between P30–50 in rats (58, 59). Thus, the reduction in 5-HT fiber juxtapositions to GnIH neurons following post-weaning social isolation may delay post-natal maturation, which may weaken the strength of existing synapses.

## Conclusion

In this study, we showed that post-weaning social isolation enhances anxiety-like behavior and an anhedonia-like phenotype that is related to altered *sert* expression in the serotonergic system. Furthermore, we demonstrated that post-weaning social isolation reduces GnIH neuronal activity and decreases 5-HT fiber juxtapositions to GnIH neurons, suggesting that serotonergic regulation may participate in GnIH signaling to accomplish normal GnRH neuronal activity and reproductive function. Although a complex molecular and neuronal mechanism is involved in post-weaning social isolation-induced reproductive dysfunction, altered serotonergic activity may be one factor that mediates GnIH–GnRH signaling in the brain. Our findings characterize the long consensus on the negative effects of post-weaning social isolation and provide insights into the neuronal and molecular mechanisms underlying the serotonergic regulation of GnIH.

## Conflict of Interest Statement

The authors declare that the research was conducted in the absence of any commercial or financial relationships that could be construed as a potential conflict of interest.
