# Supplementary material for: Early-Life Social Isolation Impairs the Gonadotropin-Inhibitory Hormone Neuronal Activity and Serotonergic System in Male Rats
Source: Front Endocrinol (Lausanne). 2015 Nov 10;6:172. doi: 10.3389/fendo.2015.00172 (PMC4639717; doi:10.3389/fendo.2015.00172)
Supplement: Supplementary file 1 [file Table_1.DOCX]

**Supplemental Table 1.** Primers Sequences used for real time PCR.

| **Primer** | **Sequence** | **product size** | **Accession #** |
| --- | --- | --- | --- |
| GnRH forward | AGC TCT GGA ACG TCT GAT TGA AG | 105bp | NM_012767 |
| GnRH reverse | TGG ATC TCA GCG TCA ATG TCA |  |  |
| GnIH forward | AGA GCA ACC TAG GAA ACG GGT GTT | 85bp | NM_023952 |
| GnIH reverse | AGG ACT GGC TGG AGG TTT CCT ATT |  |  |
|  |  |  |  |
| SERT forward | TTC TTT GCC ATC ATC TTC TTC C | 103bp | NM_013034.4 |
| SERT reverse | ACC ACG ATG AGC ACA AAC CA |  |  |
|  |  |  |  |
| TPH2 forward | TAC GGC ACC GAG CTT GAC | 84bp | NM_173839 |
| TPH2 reverse | TGG CCA CAT CCA CCA AAT AC |  |  |
|  |  |  |  |
| IMPDH2 forward | TCA AGC CAA GAA CCT CAT CGA | 146bp | NW_003809978.1 |
| IMPDH2 reverse | AGC GAC GGG CAT ACT CAG A |  |  |
|  |  |  |  |
|  |  |  |  |
